# Supplementary material for: Auranofin prevents liver fibrosis by system Xc-mediated inhibition of NLRP3 inflammasome
Source: Commun Biol. 2021 Jun 30;4:824. doi: 10.1038/s42003-021-02345-1 (PMC8245406; doi:10.1038/s42003-021-02345-1)
Supplement: Supplementary file 1 — Supplementary information [file 42003_2021_2345_MOESM1_ESM.pdf]

Supplementary Figures for

Auranofin prevents liver fibrosis by system *Xc*-mediated inhibition of NLRP3 inflammasome

Hyun Young Kim, Young Jae Choi, Sang Kyum Kim, Hyunsung Kim, Dae Won Jun, Kyungrok Yoon, Nayoun Kim, Jungwook Hwang, Young-Mi Kim, Sung Chul Lim, Keon Wook Kang*

*Corresponding author: Keon Wook Kang, Ph.D.

College of Pharmacy, Seoul National University, Gwanakro 1, Gwanak-gu, Seoul 08826, Republic of Korea; Tel: +82-2-880-7851; Fax:+82-2-872-1795; E-mail: kwkang@snu.ac.kr

Table of contents

1. **Figure S1**. Auranofin inhibits CCl4 induced liver fibrosis
2. **Figure S2**. Auranofin specifically inhibits NLRP3 inflammasome
3. **Figure S3**. Inhibitory effects of auranofin on migration and NLRP3 inflammasome in HSCs
4. **Figure S4**. Abundant antioxidants protect hepatocytes from ROS induced by auranofin
5. **Figure S5**. CD44 and xCT expression in mouse liver primary cells
6. **Full gels/blots with size markers**

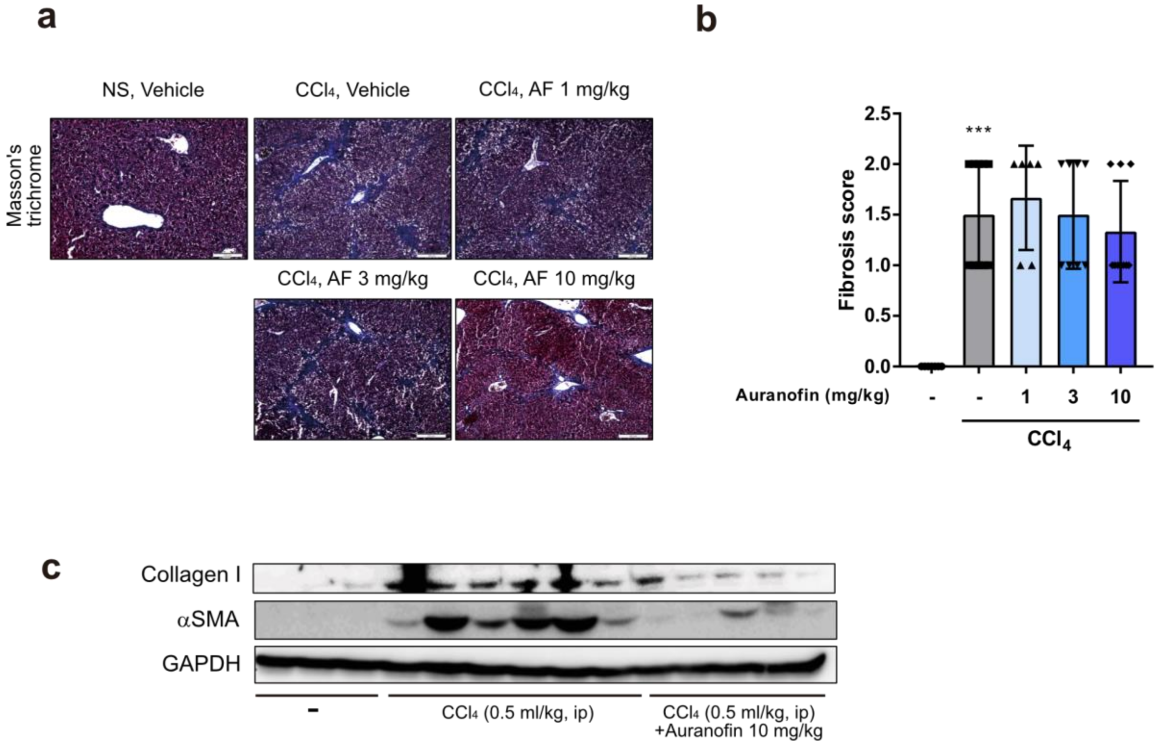

Supplementary Fig. 1. Auranofin inhibits CCl4 induced liver fibrosis.

(a) Representative Masson's Trichrome stained liver sections demonstrating collagen deposition in CCl_4 injected mice. (b) Fibrosis score assessed by liver pathologist in a blind manner. (c) Collagen 1 and αSMA expression measured by western blot analyses, n = 7 - 10 mice per group. Data are presented as mean ± SD, analyzed by one-way ANOVA followed by Tuckey's test; ***p <0.001 versus control group.

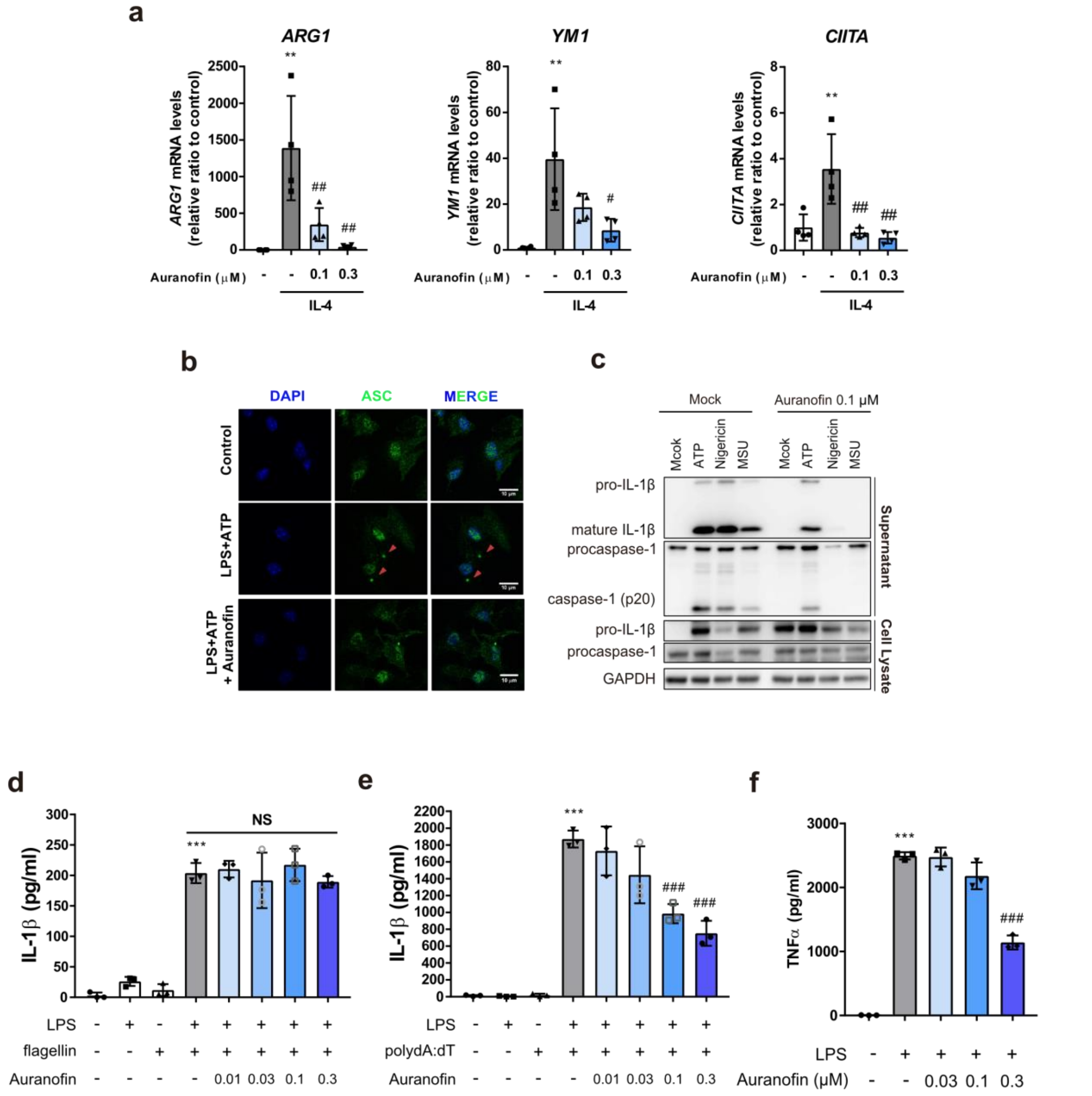

Supplementary Fig. 2. Auranofin specifically inhibits NLRP3 inflammasome

(a) Effects of auranofin on M2 polarization in BMDMs. BMDMs were treated with IL-4 (20 ng/ml) for 24 h, n=4. (b) ASC speck formation (red arrows) was observed by immunofluorescence staining in LPS-primed BMDMs treated with ATP (1 mM) and auranofin (0.1 μM). Scale bar = 10 μm (c) Effects of auranofin on ATP (1 mM), nigericin (2 μM) or 200 μg/ml monosodium urate (MSU) triggered IL-1β release in LPS-primed BMDMs. (d) Auranofin showed no effect on NLRC4 inflammasome. Activation of NLRC4 inflammasome was induced by flagellin (0.5 μg/ml) treatment, n=3. (e) Auranofin showed mild inhibitory effect on AIM2 inflammasome. Activation of AIM2 inflammasome was induced by 1 μg/ml poly(dA:dT) transfection, n=3. (f) TNFα secretion was measured after incubation of BMDMs with LPS (100 ng/ml) for 6 h, n=3. Data are presented as mean ± SD (a, d, e and f), analyzed by one-way ANOVA followed by Tuckey's test; **$p<0.01$ and ***$p<0.001$, compared to control; #$p<0.05$, ##$p<0.01$ and ###$p<0.001$, NS; not significant, compared to inflammasome-induced group.

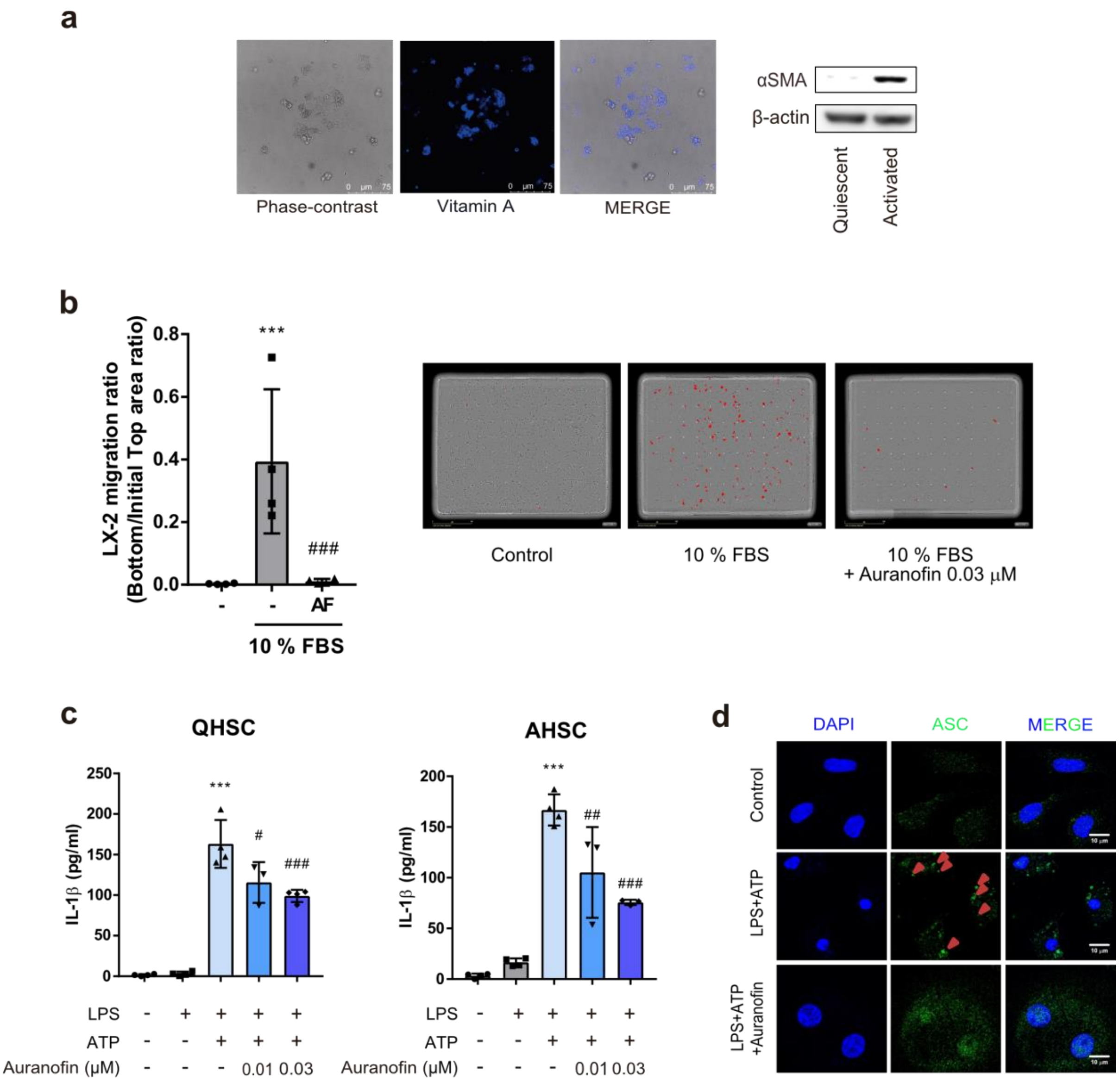

Supplementary Fig. 3. Inhibitory effects of auranofin on migration and NLRP3 inflammasome in HSCs.

(a) Fluorescence microscopy for the detection of autofluorescence of vitamin A in quiescent HSC isolated from mouse liver. 7 days after activation of isolated quiescent HSC on plastic plate, αSMA protein levels were evaluated using immunoblotting. (b) Migration of LX-2 cells was measured by Incucyte® chemotaxis assay, n=4. Data are presented as mean ± SD, analyzed by one-way ANOVA followed by Tuckey's test; ***p <0.001 compared to control group; ###p<0.01 compared to migrated cell counts in FBS containing media. AF; Auranofin 0.03 μM. (c) Effects of auranofin on ATP triggered IL-1β release of LPS-primed HSCs. Quiescent and activated HSCs were primed with LPS (100 ng/mL) for 4 h, followed by exposure to ATP (5 mM) and auranofin for 1 h, n=4. Data are presented as mean ± SD, analyzed by one-way ANOVA followed by Tuckey's test; ***p <0.001, compared to control; #p<0.05, ##p<0.01 and ###p <0.001, compared to NLRP3 inflammasome-induced group. (d) ASC speck formation (red arrows) in LPS-primed HSCs treated with ATP (5 mM) and auranofin for 1 h was determined by immunofluorescence staining.

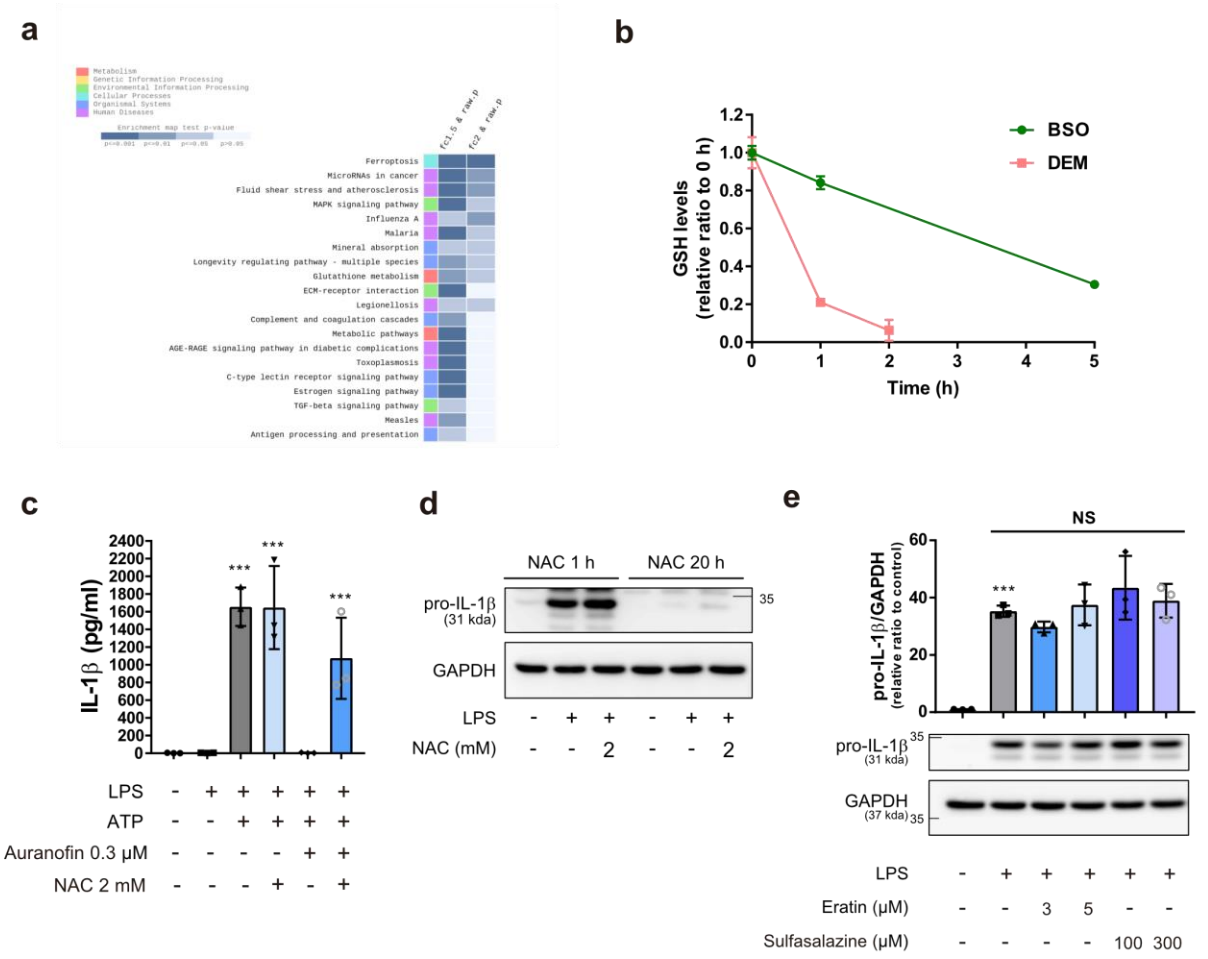

Supplementary Fig. 4. Abundant antioxidants protect hepatocytes from ROS induced by auranofin

(a) KEGG pathways analysis after RNA sequencing. RNA samples isolated from primary hepatocytes after exposure to 1 μM auranofin for 12 h were used. (b) Time-dependent intracellular GSH levels in BMDMs followed by treatment of GSH-depleting reagents, n=3. BSO; buthionine sulfoximine 100 μM, DEM; diethylmaleate 300 μM. (c) ATP triggered IL-1β release by NLRP3 inflammasome was determined by ELISA in supernatants of LPS-primed BMDM after cotreatment of NAC with auranofin, n=3. (d) The protein expression level of pro-IL-1β was not altered in LPS-primed macrophages 1 h or 20 h after NAC (2 mM) treatment. (e) Effects of xCT inhibitors, erastin and sulfasalazine on pro-IL-1β expression in BMDM. LPS-primed BMDMs were exposed to erastin or sulfasalazine for 1 h, n=3. (b, c and e) Data are presented as mean ± SD, analyzed by one-way ANOVA followed by Tuckey's test; ***p <0.001, NS; not significant compared to LPS treated BMDMs.

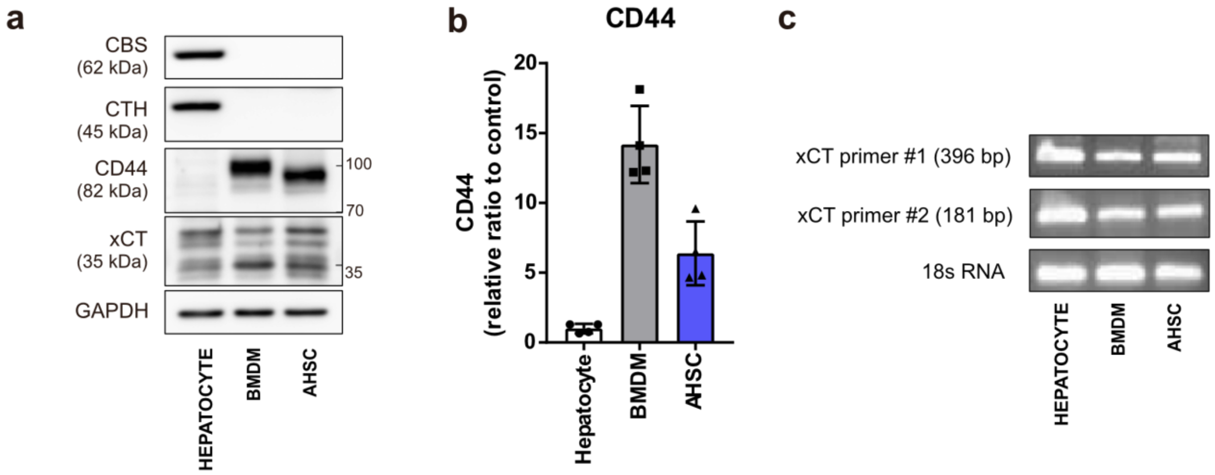

Supplementary Fig. 5. CD44 and xCT expression in mouse liver primary cells
(a) Protein expression level of transsulfuration enzymes; cystathionine β-synthase (CBS), cystathionine γ-lyase (CTH), CD44 and xCT in primary hepatocytes, BMDMs and activated HSCs. (b and c) Relative mRNA expression level for CD44 and xCT were evaluated by qPCR and conventional PCR, respectively. Data of CD44 expression are presented as mean ± SD, n=4.

Full gels/blots with size markers

Fig. 1d

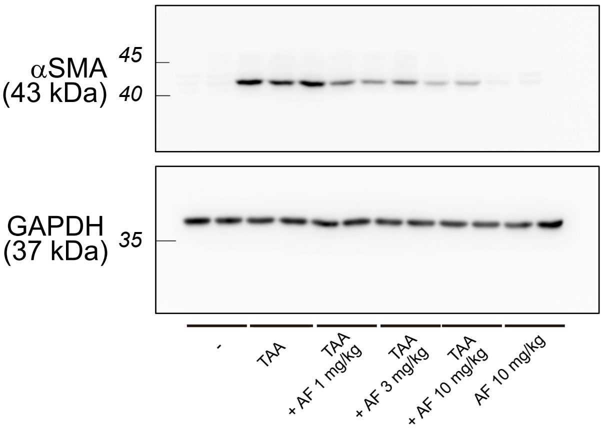

Fig. 2a

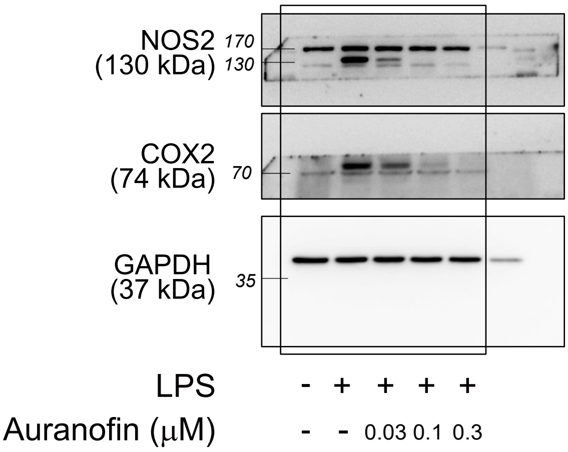

Fig. 2d

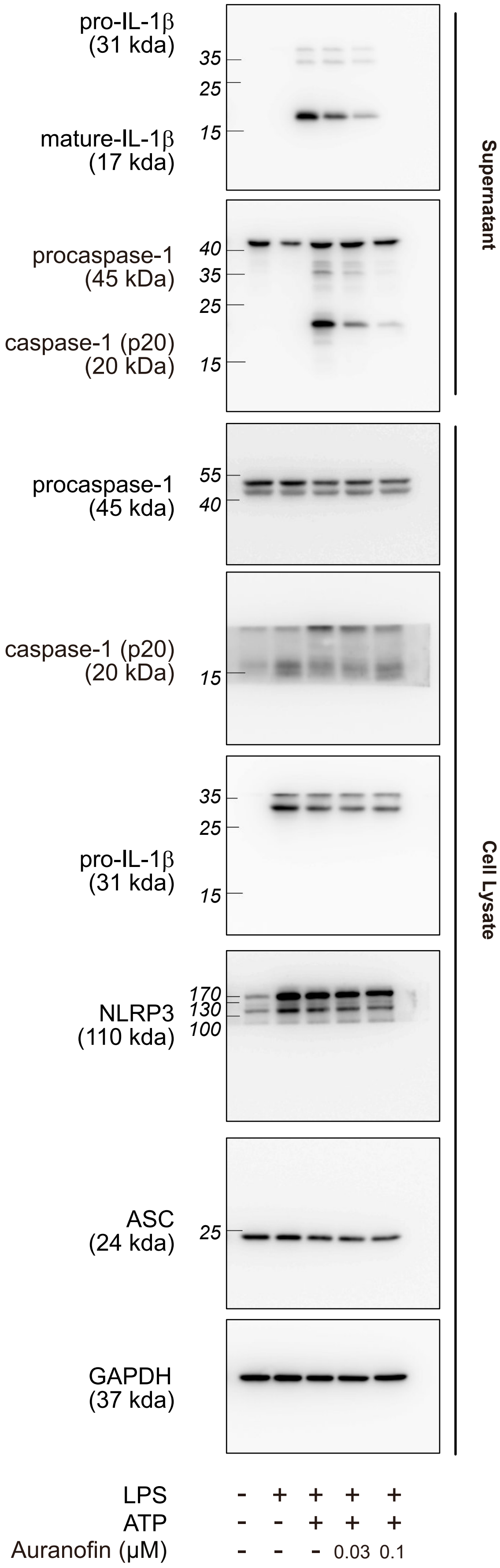

Fig. 2b

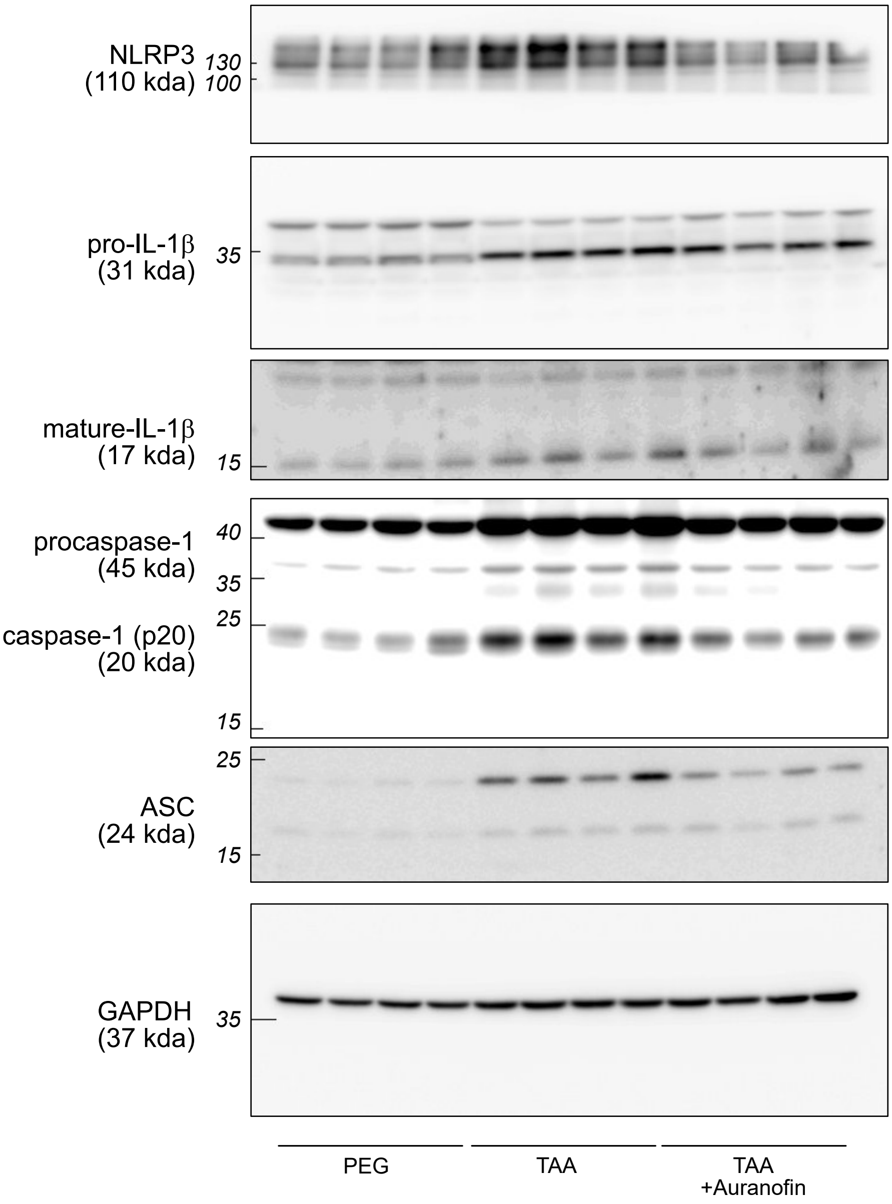

Fig. 3b

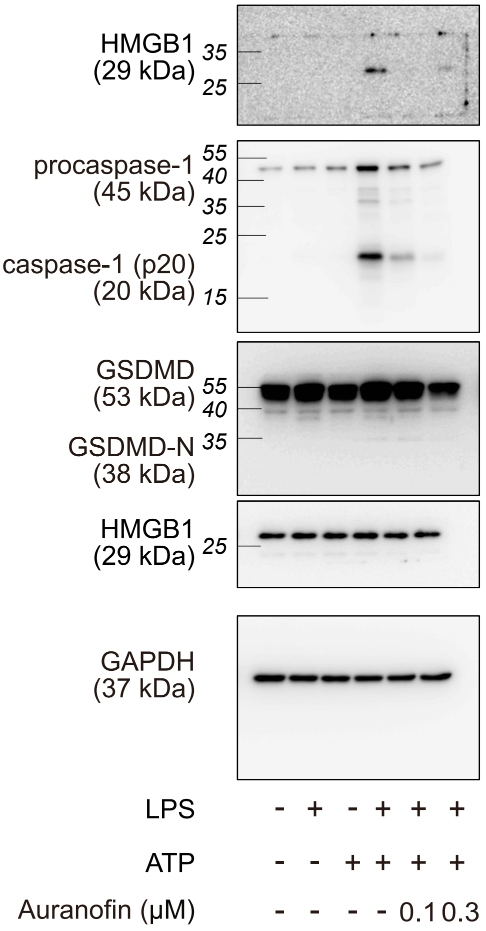
HMGB1
(29 kDa)
35
25
procaspase-1
(45 kDa)
caspase-1 (p20)
(20 kDa)
55
40
35
25
15
GSDMD
(53 kDa)
GSDMD-N
(38 kDa)
55
40
35
HMGB1
(29 kDa)
25
GAPDH
(37 kDa)
LPS - + - + + +
ATP - - + + + +
Auranofin (μM) - - - - 0.1 0.3

Fig. 3d

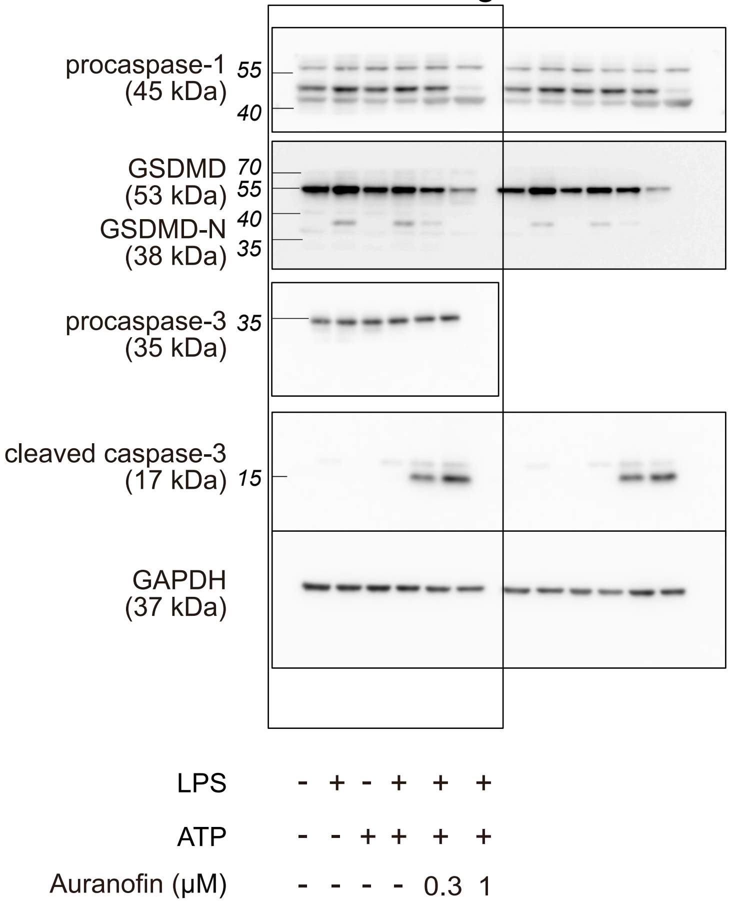
procaspase-1
(45 kDa)
55
40
GSDMD
(53 kDa)
GSDMD-N
(38 kDa)
70
55
40
35
procaspase-3
(35 kDa)
35
cleaved caspase-3
(17 kDa)
15
GAPDH
(37 kDa)
LPS - + - + + +
ATP - - + + + +
Auranofin (μM) - - - - 0.3 1

Fig. 4c

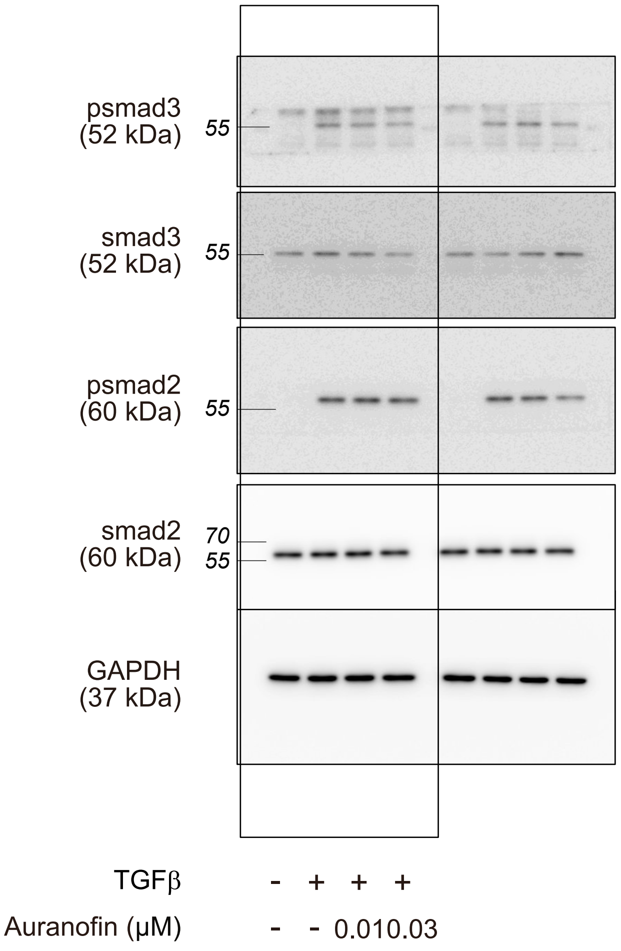

psmad3
(52 kDa)
55
smad3
(52 kDa)
55
psmad2
(60 kDa)
55
smad2
(60 kDa)
70
55
GAPDH
(37 kDa)
TGFβ
- + + +
Auranofin (μM)
- - 0.01 0.03

Fig. 5g

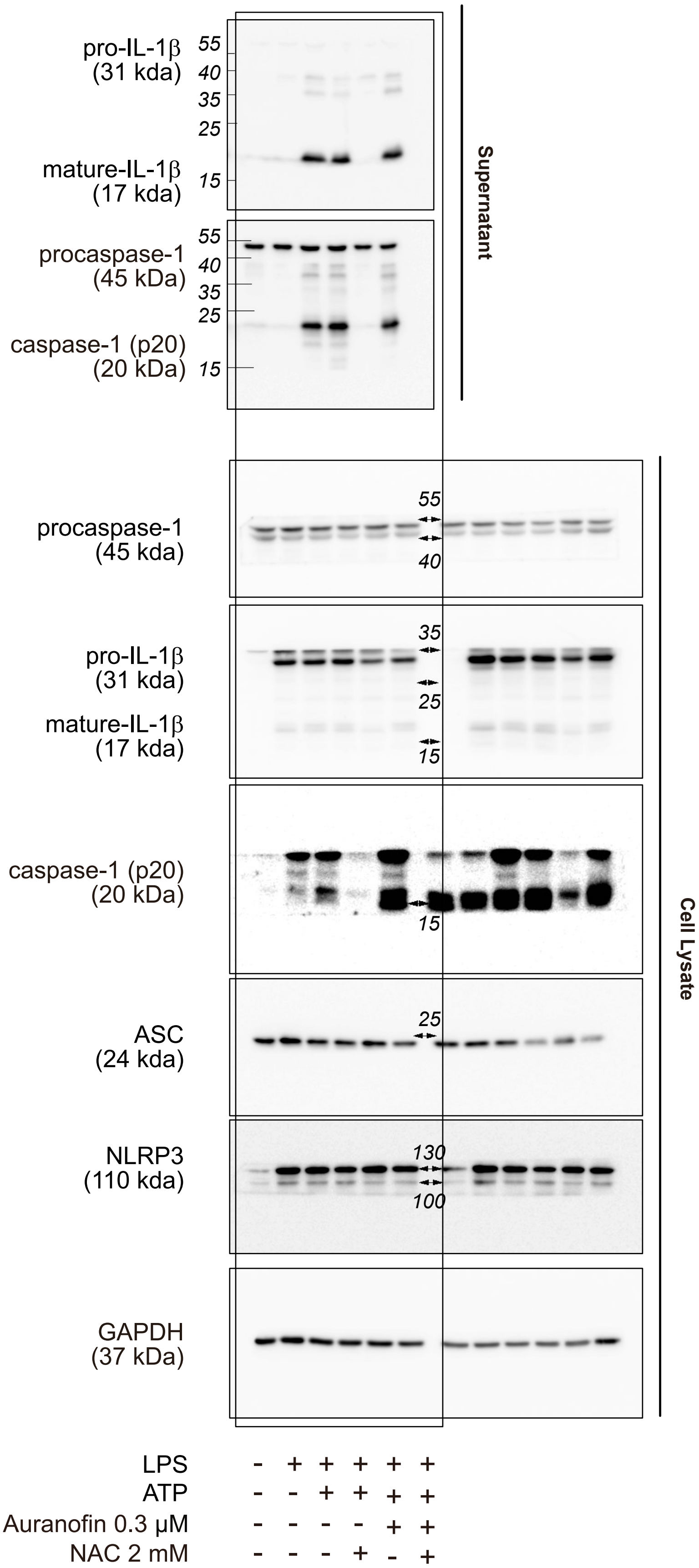

Fig. 5h

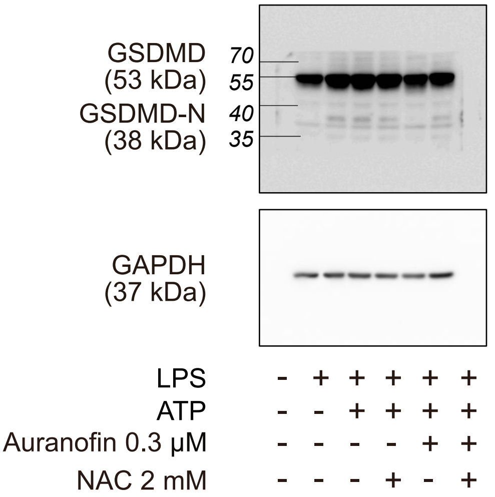

Fig. 5i

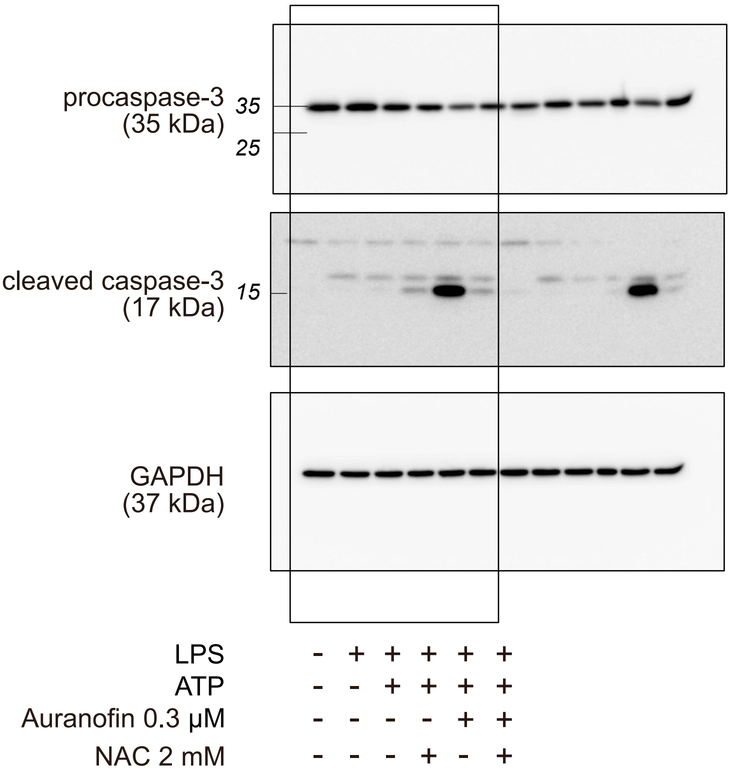

Fig. 6d

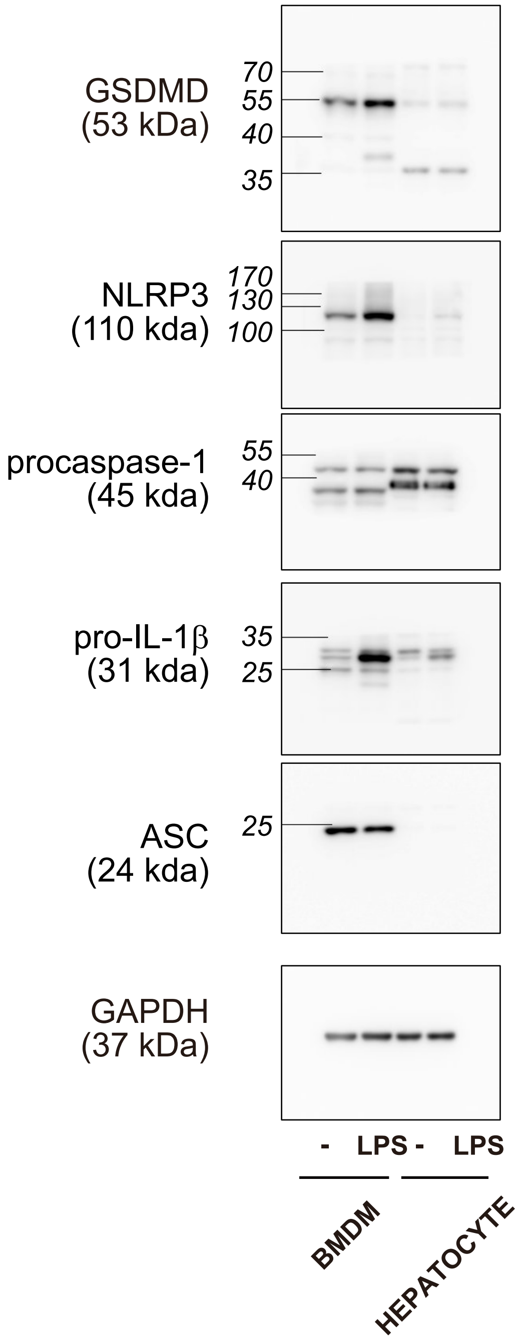
GSDMD
(53 kDa)
70
55
40
35
NLRP3
(110 kda)
170
130
100
procaspase-1
(45 kda)
55
40
pro-IL-1β
(31 kda)
35
25
ASC
(24 kda)
25
GAPDH
(37 kDa)
-
LPS
-
LPS
BMDM
HEPATOCYTE

Fig. 7a

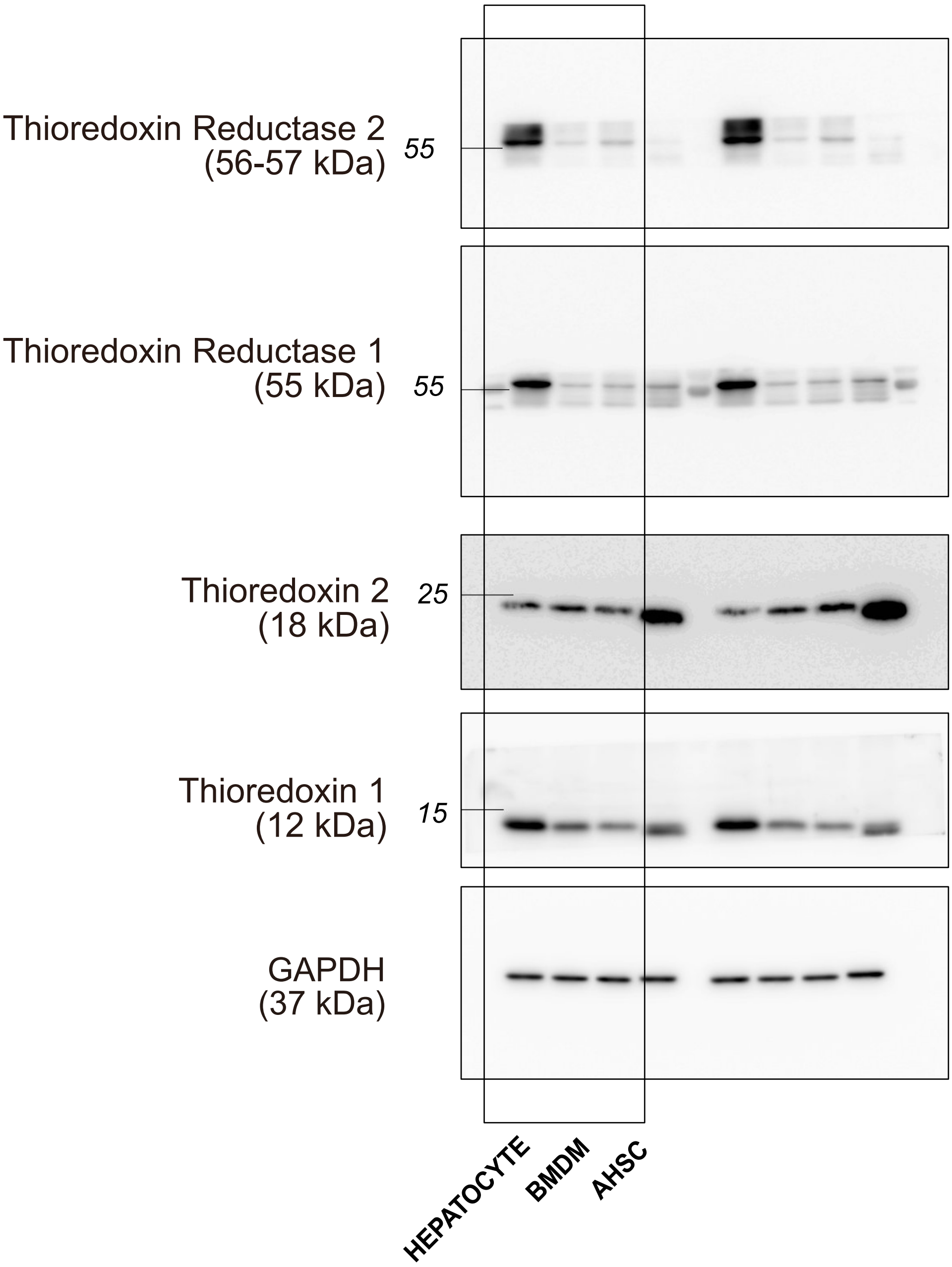
Thioredoxin Reductase 2
(56-57 kDa)
55
Thioredoxin Reductase 1
(55 kDa)
55
Thioredoxin 2
(18 kDa)
25
Thioredoxin 1
(12 kDa)
15
GAPDH
(37 kDa)
HEPATOCYTE
BMDM
AHSC
