# Supplementary material for: Auranofin prevents liver fibrosis by system Xc-mediated inhibition of NLRP3 inflammasome
Source: Commun Biol. 2021 Jun 30;4:824. doi: 10.1038/s42003-021-02345-1 (PMC8245406; doi:10.1038/s42003-021-02345-1)
Supplement: Supplementary file 2 — Description of Additional Supplementary Files [file 42003_2021_2345_MOESM2_ESM.pdf]

Description of Additional Supplementary Files

File Name: Supplementary Data 1
Description: data sets and exact pvalues for the graphs presented in the main figures.
