# Supplementary material for: Auranofin prevents liver fibrosis by system Xc-mediated inhibition of NLRP3 inflammasome
Source: Commun Biol. 2021 Jun 30;4:824. doi: 10.1038/s42003-021-02345-1 (PMC8245406; doi:10.1038/s42003-021-02345-1)
Supplement: Supplementary file 4 — Reporting Summary [file 42003_2021_2345_MOESM4_ESM.pdf]

nature research

Corresponding author(s): Keon Wook Kang

Last updated by author(s): Jun 7, 2021

Reporting Summary

Nature Research wishes to improve the reproducibility of the work that we publish. This form provides structure for consistency and transparency in reporting. For further information on Nature Research policies, see our Editorial Policies and the Editorial Policy Checklist.

Statistics

For all statistical analyses, confirm that the following items are present in the figure legend, table legend, main text, or Methods section.

n/a	Confirmed	
☐	☒	The exact sample size (*n*) for each experimental group/condition, given as a discrete number and unit of measurement
☐	☒	A statement on whether measurements were taken from distinct samples or whether the same sample was measured repeatedly
☐	☒	The statistical test(s) used AND whether they are one- or two-sided<br>*Only common tests should be described solely by name; describe more complex techniques in the Methods section.*
☐	☒	A description of all covariates tested
☐	☒	A description of any assumptions or corrections, such as tests of normality and adjustment for multiple comparisons
☐	☒	A full description of the statistical parameters including central tendency (e.g. means) or other basic estimates (e.g. regression coefficient) AND variation (e.g. standard deviation) or associated estimates of uncertainty (e.g. confidence intervals)
☐	☒	For null hypothesis testing, the test statistic (e.g. *F*, *t*, *r*) with confidence intervals, effect sizes, degrees of freedom and *P* value noted<br>*Give P values as exact values whenever suitable.*
☒	☐	For Bayesian analysis, information on the choice of priors and Markov chain Monte Carlo settings
☒	☐	For hierarchical and complex designs, identification of the appropriate level for tests and full reporting of outcomes
☒	☐	Estimates of effect sizes (e.g. Cohen's *d*, Pearson's *r*), indicating how they were calculated

Our web collection on statistics for biologists contains articles on many of the points above.

Software and code

Policy information about availability of computer code

Data collection	No unpublished software was used.
Data analysis	Data was analyzed using GraphPad Prism (version 7.0) and NIH Image J (1.8.0_112).

For manuscripts utilizing custom algorithms or software that are central to the research but not yet described in published literature, software must be made available to editors and reviewers. We strongly encourage code deposition in a community repository (e.g. GitHub). See the Nature Research guidelines for submitting code & software for further information.

Data

Policy information about availability of data

All manuscripts must include a data availability statement. This statement should provide the following information, where applicable:

- Accession codes, unique identifiers, or web links for publicly available datasets
- A list of figures that have associated raw data
- A description of any restrictions on data availability

The data that support the findings of this study are available from the lead corresponding author (K.W.K) on reasonable request.

Field-specific reporting

Life sciences study design

All studies must disclose on these points even when the disclosure is negative.

Sample size	In vitro assays (e.g. Western blot, ELISA, cystine uptake assay) were repeated at least three times.
Data exclusions	N/A
Replication	All attempts at replication were successful.
Randomization	N/A
Blinding	For the measurement of fibrosis score in animal experiments, we masked the name of the samples and the quantifications were perfomed by an expert.

Reporting for specific materials, systems and methods

We require information from authors about some types of materials, experimental systems and methods used in many studies. Here, indicate whether each material, system or method listed is relevant to your study. If you are not sure if a list item applies to your research, read the appropriate section before selecting a response.

Materials & experimental systems

n/a	Involved in the study	
☐	☒	Antibodies
☐	☒	Eukaryotic cell lines
☒	☐	Palaeontology and archaeology
☐	☒	Animals and other organisms
☒	☐	Human research participants
☒	☐	Clinical data
☒	☐	Dual use research of concern

Methods

n/a	Involved in the study	
☒	☐	ChIP-seq
☐	☒	Flow cytometry
☒	☐	MRI-based neuroimaging

Antibodies

Antibodies used

Antibody name (Species, Applications, Cat. NO., LOT. NO., Clone name/number, Company)
[Primary antibody]
anti-aSMA (rabbit, WB, ab32575,-,-, Abcam)
anti-aSMA (mouse, IF, A5228,-,-, Sigma)
anti-NOS2 (rabbit, WB, sc-650,-,-, Santa cruz)
anti-COX2 (rabbit, WB, 4842S,-,-, Cell Signaling Technology)
anti-GAPDH (mouse, WB, CB1001,-,-, Millipore)
anti-NLRP3 (rabbit, WB, AG-20B-0014-C100,-,-, Adipogen)
anti-IL1b (rabbit, WB, ab9722,-,-, Abcam)
anti-caspase1 (mouse, WB, sc-56036,-,-, Santa Cruz)
anti-caspase-1, cleaved (mouse, WB, AG-20B-0042-C100,-,-, Adipogen)
anti-ASC (rabbit, WB, AG-25B-0006-C100,-,-, Adipogen)
anti-ASC (mouse, IF, sc-271054,-,-, Santa Cruz)
anti-GSDMDC1 (mouse, WB, sc-393656,-,-, Santa Cruz)
anti-HMGB1 (rabbit, WB, 3935S,-,-, Cell Signaling Technology)
anti-caspase-3 (rabbit, WB, 9662S,-,-, Cell Signaling Technology)
anti-caspase-3, cleaved (rabbit, WB, 9664S,-,-, Cell Signaling Technology)
anti-psmad3 (rabbit, WB, 9520S,-,-, Cell Signaling Technology)
anti-smad3 (rabbit, WB, 9523S,-,-, Cell Signaling Technology)
anti-psmad2 (rabbit, WB, 3108S,-,-, Cell Signaling Technology)
anti-smad2 (rabbit, WB, 5339S,-,-, Cell Signaling Technology)
anti-TrxR2 (mouse, WB, sc-376868,-,-, Santa Cruz)
anti-Trx2 (rabbit, WB, sc-50336,-,-, Santa Cruz)
anti-TrxR1 (mouse, WB, sc-28321,-,-, Santa Cruz)
anti-Trx1 (rabbit, WB, 2429S,-,-, Cell Signaling Technology)
anti-xCT (rabbit, WB/IF/IHC, NB300-318,-,-, Novus)
anti-CD44 (rabbit, WB/IF, ab157107,-,-, Abcam)
anti-CBS (mouse, WB, sc-133154,-,-, Santa Cruz)
anti-CTH (mouse, H00001491-M01, -,-, Abnova)
anti-F4/80 (mouse, IHC, sc-377009,-,-, Santa Cruz)

[Secondary antibody]
Anti-mouse IgG, HRP-linked Antibody (mouse, WB, 7076S,-,-, Cell Signaling Technology)
Anti-rabbit IgG, HRP-linked Antibody (rabbit, WB, 7074S,-,-, Cell Signaling Technology)

Validation

Format:
Antibody name (Species, Applications, Cat. NO., LOT. NO., Clone name/number, Company)
[Primary antibody]
anti-aSMA (rabbit, WB, ab32575,-,-, Abcam)
Species reactivity: Mouse, Rat
Application: WB, IHC-P, Flow Cyt, ICC/IF
Citation: PMID 31746423, PMID 31704583

anti-aSMA (mouse, IF, A5228,-,-, Sigma)
Species reactivity: Mouse, Human, Frog, Sheep, Chicken, Goat, Bovine, Rat, Guinea pig, Canine, Rabbit, Snake
Application: WB, IHC, ICC/IF, Microarray
Citation: PMID 21681858

anti-NOS2 (rabbit, WB, sc-650,-,-, Santa cruz)
Species reactivity: Mouse, Rat
Application: WB, IP, IF
Citation: PMID 27239349, PMID 26838169

anti-GAPDH (mouse, WB, CB1001,-,-, Millipore)
Species reactivity: Canine, Chicken, Fish, Frog, Human, Mouse, Porcine, Rabbit
Application: ELISA, WB, IHC
Citation: PMID 16005468

anti-NLRP3 (rabbit, WB, AG-20B-0014-C100,-,-, Adipogen)
Species reactivity: Human, Mouse
Application: WB, IF, IP, IHC, ChIP
Citation: PMID 21994456

anti-IL1b (rabbit, WB, ab9722,-,-, Abcam)
Species reactivity: Recombinant fragment
Application: Sandwich ELISA, WB
Citation: PMID 31539804, PMID 31931832

anti-caspase1 (mouse, WB, sc-56036,-,-, Santa Cruz)
Species reactivity: Human, Mouse, Rat
Application: WB, IP, IF
Citation: PMID 31432144, PMID 31327667

anti-caspase-1, cleaved (mouse, WB, AG-20B-0042-C100,-,-, Adipogen)
Species reactivity: Mouse
Application: WB, IHC, IP
Citation: PMID: 22444631 PMID: 23809162

anti-ASC (rabbit, WB, AG-25B-0006-C100,-,-, Adipogen)
Species reactivity: Mouse, Human
Application: WB, IHC, IP
Citation: PMID 24952504

anti-ASC (mouse, IF, sc-271054,-,-, Santa Cruz)
Species reactivity: Mouse, Human, Rat
Application: WB, IHC, IP, IF, Solid Phase ELISA
Citation: PMID 23209696

anti-GSDMDC1 (mouse, WB, sc-393656,-,-, Santa Cruz)
Species reactivity: Mouse, Rat
Application: WB, IF, IP, WB, Solid Phase ELISA
Citation: PMID 31097341, PMID 31145977

anti-HMGB1 (rabbit, WB, 3935S,-,-, Cell Signaling Technology)
Species reactivity: Mouse, Human, Rat, Monkey
Application: WB, IF
Citation: PMID 31545489

anti-caspase-3 (rabbit, WB, 9662S,-,-, Cell Signaling Technology)
Species reactivity: Mouse, Human, Rat, Monkey

Application: WB, IP, IHC
Citation: PMID 31971852

anti-caspase-3, cleaved (rabbit, WB, 9664S,-,-, Cell Signaling Technology)
Species reactivity: Mouse, Human, Rat, Monkey
Application: WB, IP, IHC, IF, F
Citation: PMID 31854220

anti-psmad3 (rabbit, WB, 9520S,-,-, Cell Signaling Technology)
Species reactivity: Mouse, Human, Rat
Application: WB, IP, ChIP
Citation: PMID 32187849

anti-smad3 (rabbit, WB, 9523S,-,-, Cell Signaling Technology)
Species reactivity: Mouse, Human, Rat, Monkey
Application: WB, IP, IF, F, ChIP
Citation: PMID 32477928

anti-psmad2 (rabbit, WB, 3108S,-,-, Cell Signaling Technology)
Species reactivity: Mouse, Human, Rat, Mink
Application: WB
Citation: PMID 32434697

anti-smad2 (rabbit, WB, 5339S,-,-, Cell Signaling Technology)
Species reactivity: Mouse, Human, Rat, Monkey
Application: WB, IP, IF, F, ChIP
Citation: PMID 32269624

anti-TrxR2 (mouse, WB, sc-376868,-,-, Santa Cruz)
Species reactivity: Mouse, Rat
Application: WB, IP, IF, IHC, Solid Phase ELISA
Citation: PMID 31078905

anti-Trx2 (rabbit, WB, sc-50336,-,-, Santa Cruz)
Species reactivity: Mouse, Rat, Human
Application: WB, IP, IF, IHC, Solid Phase ELISA
Citation: PMID 30773462

anti-TrxR1 (mouse, WB, sc-28321,-,-, Santa Cruz)
Species reactivity: Mouse, Rat, Human
Application: WB, IP, IF, IHC, Solid Phase ELISA
Citation: PMID 30799286

anti-Trx1 (rabbit, WB, 2429S,-,-, Cell Signaling Technology)
Species reactivity: Mouse, Rat, Human
Application: WB, IHC
Citation: PMID: 27807255

anti-xCT (rabbit, WB/IF/IHC, NB300-318,-,-, Novus Biologicals)
Species reactivity: Mouse, Rat, Human
Application: WB, F, ICC/IF, IHC, IHC-P, Dual ISH-IHCC
Citation: PMID 31264167

anti-CD44 (rabbit, WB/IF, ab157107,-,-, Abcam)
Species reactivity: Mouse, Rat, Human
Application: ICC/IF, WB, IP, IHC-P
Citation: PMID 32398747

anti-CBS (mouse, WB, sc-133154,-,-, Santa Cruz)
Species reactivity: Mouse, Rat, Human
Application: WB, IP, IHC, Solid Phase ELISA
Citation: PMID 31111947

anti-CTH (mouse, H00001491-M01, -,-, Abnova)
Species reactivity: Mouse, Guinea pig, Human
Application: WB, Sandwich ELISA
Citation: PMID 26047341

anti-F4/80 (mouse, IHC, sc-377009,-,-, Santa Cruz)
Species reactivity: Mouse, Rat, Human
Application: WB, IP, IHC, Solid Phase ELISA
Citation: PMID 25136608

[Secondary antibody]
Anti-mouse IgG, HRP-linked Antibody (mouse, WB, 7076S,-,-, Cell Signaling Technology)
Species reactivity: Mouse
Application: WB
Citation: PMID 23849170

Anti-rabbit IgG, HRP-linked Antibody (rabbit, WB, 7074S,-,-, Cell Signaling Technology)
Species reactivity: Rabbit
Application: WB
Citation: PMID 31324602

Eukaryotic cell lines

Policy information about cell lines

Cell line source(s)	LX-2
Authentication	LX-2 Cell line was obtained from Scott L. Friedman, M.D. (PMID 21837750)
Mycoplasma contamination	LX-2 cell line was tested negative for mycoplasma contamination.
Commonly misidentified lines (See ICLAC register)	N/A

Animals and other organisms

Policy information about studies involving animals; ARRIVE guidelines recommended for reporting animal research

Laboratory animals	Mouse, C57BL/6, Male, 8 to 12 weeks old
Wild animals	N/A
Field-collected samples	N/A
Ethics oversight	IACUC (Institutional Animal Care and Use Committee; SNU-171127-2, SNU-181105-6-1 and SNU-140930-5) in Seoul National University

Note that full information on the approval of the study protocol must also be provided in the manuscript.

Flow Cytometry

Plots

Confirm that:

- [x] The axis labels state the marker and fluorochrome used (e.g. CD4-FITC).
- [x] The axis scales are clearly visible. Include numbers along axes only for bottom left plot of group (a 'group' is an analysis of identical markers).
- [x] All plots are contour plots with outliers or pseudocolor plots.
- [x] A numerical value for number of cells or percentage (with statistics) is provided.

Methodology

Sample preparation	Cells were harvested by rubber policeman and washed with PBS
Instrument	FACS Calibur flow cytometer 2 (BD science, San Jose, CA, USA)
Software	BD CellQuest™ Pro Software
Cell population abundance	Living cells in PBS were gated by FSC/SSC using CellQuest™ Pro Software
Gating strategy	Cells were gated by FSC/SSC

- [ ] Tick this box to confirm that a figure exemplifying the gating strategy is provided in the Supplementary Information.
